# A marked elevation in serum creatinine/cystatin C ratio may indicate pseudo-acute kidney injury due to urinary ascites: a case report and literature review

**DOI:** 10.1186/s12882-023-03289-w

**Published:** 2023-08-21

**Authors:** Ran Jiang, Yumin Huang, Ming Zeng, Changying Xing, Huijuan Mao, Buyun Wu

**Affiliations:** Department of Nephrology, Jiangsu Province Hospital, The First Affiliated Hospital of Nanjing Medical University, Nanjing, 210029 China

**Keywords:** Pseudo-acute kidney injury, Urinary ascites, Spontaneous bladder rupture, Serum creatinine, Serum cystatin C

## Abstract

**Background:**

Urinary ascites represents a scarcely observed pseudo-acute kidney injury in clinical settings. Protracted or missed diagnosis may hold grave ramifications for patient outcomes.

**Case presentation:**

We reported a case involving an elderly female patient experiencing pseudo-acute kidney injury accompanied by ascites, wherein her renal dysfunction persisted despite medical intervention and hemodialysis. Urinary ascites was identified via a methylene blue test and by contrasting creatinine levels in serum and ascites. This patient’s kidney function was multiple typified by a marked elevation in serum creatinine/Cystatin C ratio (> 2 L/dL), potentially serving as a clue for the clinical diagnosis of pseudo-acute kidney injury engendered by urinary ascites.

**Conclusions:**

This case suggested the potential diagnostic value of an asynchronous increase in serum creatinine and serum CysC (or an increased ratio of blood creatinine to blood CysC) in patients with pseudo-acute kidney injury.

## Background

Urinary ascites denotes the aggregation of fluid within the abdominal region resulting from a ruptured bladder, permitting the passage of urine via a fissure in the bladder wall and into the peritoneal cavity [1]. Previous literature suggests that the incidence of the spontaneous bladder rupture is about 0.002% [2]. From a clinical standpoint, urinary ascites frequently manifests as acute kidney injury (AKI) and ascites [3]. Diagnostic confirmation can be obtained through imaging that shows the rupture location, bladder infusion dye examination, or an elevated ascitic creatinine/serum creatinine ratio [4]. Failure to expeditiously identify this condition can have a grave impact on a patient’s prognosis. We present a case involving an 85-year-old female experiencing a spontaneously ruptured bladder and obstructive uropathy, presenting with a deceptive AKI. Indications of this rare AKI subtype were revealed through elevated serum creatinine and stable cystatin C levels. The following report provides details of this case.

## Case presentation

**Fig. 1:**
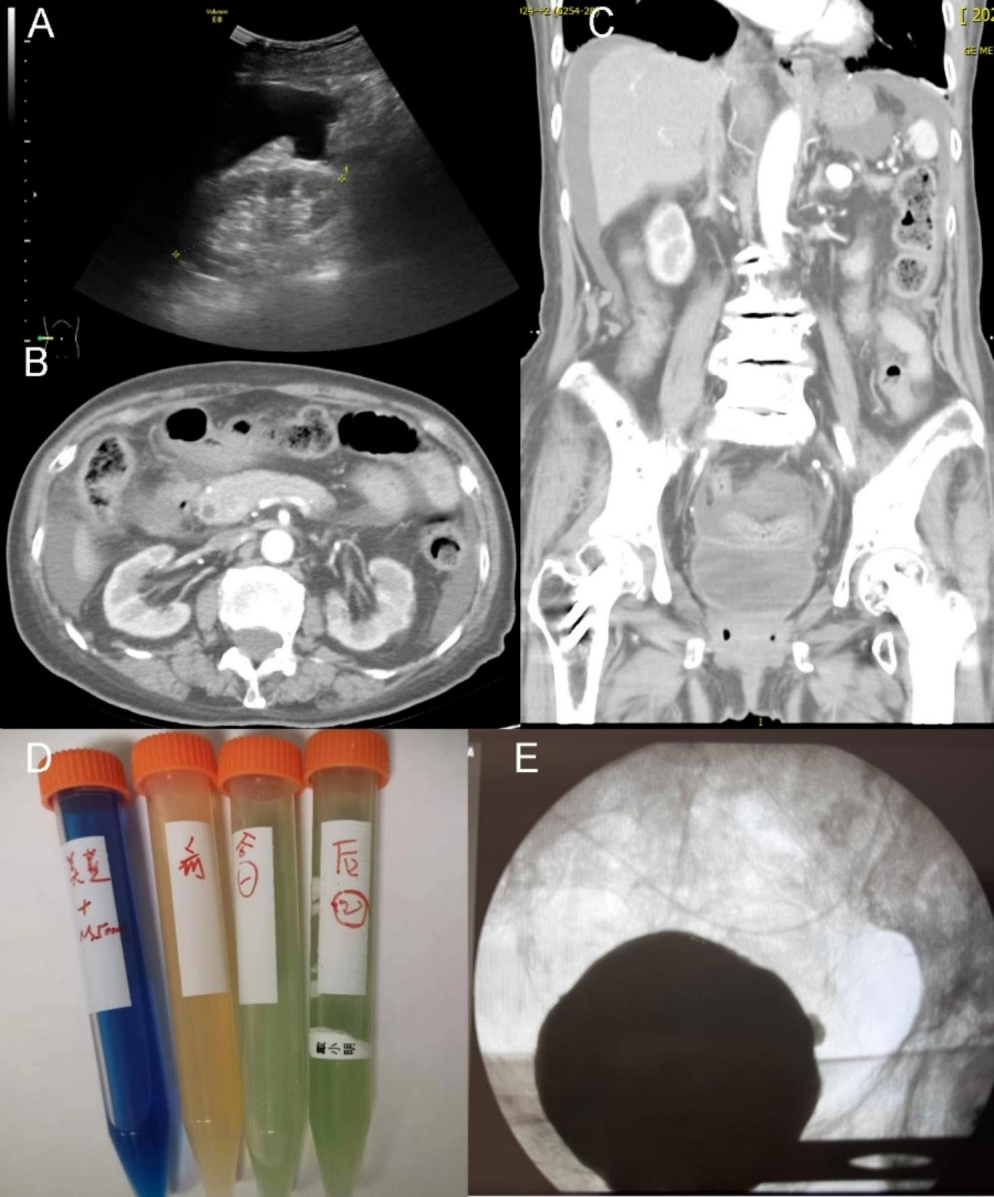
The main results for the examinations in the patient. Note: The kidney ultrasound revealed the presence of ascites and the absence of hydronephrosis in emergency room (**A**). Further contrasted computed tomography scan confirmed no hydronephrosis (**B**) and the presence of urine in the bladder (**C**). The methylene blue test conducted on the day of catheter placement yielded positive results (**D**). Arranged from left to right, the fluids exhibited the colors blue (methylene blue solution), yellow (ascitic fluid before the test), light green (ascitic fluid one hour after the test), and green (ascitic fluid two hours after the test), respectively. Subsequent to three days of indwelling Foley catheterization, a contrast-enhanced fluid containing a radiopaque agent was infused into the bladder via the catheter, and cystography examination revealed no evidence of leakage (**E**)

In August 2022, an 85-year-old woman was admitted to our facility, presenting with abdominal discomfort for three days and anuria for two days. The abdominal pain, predominantly occurring in the upper region, lacked an apparent cause and was unaccompanied by chills, fever, nausea, vomiting, diarrhea, or back discomfort. Emergency blood tests indicated a white blood cell count of 7,960/µl, an 83.7% neutrophil prevalence, a 112 g/L hemoglobin concentration, a 218,000/µl platelet count, a blood creatinine level of 1.4 mg/dL, and a 133 mmol/L blood sodium level. A renal ultrasound and whole abdomen CT scans revealed fluid accumulation in the abdominal and pelvic cavities (Fig. 1.A-B). In order to exclude mesenteric arterial thrombosis as the cause, a CTA scan was conducted, revealing no discernible filling defects within the mesenteric arteries. Other findings included thickened, enhanced colonic wall in right colic flexure and indistinct bladder filling (Fig. 1.C). The patient received symptomatic treatment of lansoprazole for gastric protection and cefotaxime for potential peritonitis. Upon returning home, the patient exhibited anuria, accompanied by nausea, vomiting, and lower right abdominal pain. Subsequent blood tests indicated a white blood cell count of 9,470/µl, an 80.0% neutrophil prevalence, a 118 g/L hemoglobin concentration, a blood creatinine level of 4.16 mg/dL, and a 132 mmol/L blood sodium level. Consequently, the patient was admitted to the nephrology department for urgent care.

The patient’s medical history included a total hysterectomy with adnexectomy for endometrial cancer in 1992, followed by radiotherapy and chemotherapy. Additional diagnoses encompassed radiation enteritis, gastrointestinal bleeding, colonic polyps, urinary tract infection, urinary retention, and AKI (necessitating dialysis). The medications of the patient include Tamsulosin, Verapamil. Half a year prior to admission, the patient’s blood creatinine level was 0.89 mg/dL. Upon admission, a physical examination revealed a body temperature of 36.5℃, a respiratory rate of 16 breaths/min, a pulse rate of 80 beats/min, and positive signs of abdominal distention, mild tenderness, rebound tenderness in the upper and lower abdomen, and slight abdominal muscular tension. Pitting edema was observed in both lower extremities.

Upon admission, the patient promptly received emergency hemodialysis, and subsequent blood tests revealed a creatinine level of 5.86 mg/dL and a cystatin C level of 1.73 mg/dL (Table 1), along with a C-reactive protein level of 244 mg/L, and a procalcitonin level of 0.33 ng/mL. The preliminary diagnosis indicated stage 3 acute kidney injury, potentially stemming from contrast media exposure or infection. The individual’s abdominal discomfort and elevated C-reactive protein hinted at sepsis or acute enteritis, prompting treatment with Imipenem and Cilastatin for anti-infective therapy, as well as intermittent hemodialysis. Following a ten-day treatment course, the patient’s C-reactive protein levels significantly decreased, yet anuria persisted, and ascites continued to amass. Abdominal paracentesis was performed, revealing a yellow-clear fluid with minimal levels of white and red blood cells, as well as total protein, suggestive of an exudative nature. An abdominal drainage tube was placed, with 500–600 mL of ascites drained daily to alleviate abdominal distension.

**Table 1:** Changes of laboratory data and urine output in this patient

Date	Admission	Day 1	Day 10	Day 22	Day 23	Day 26
**Laboratory data**						
Blood white blood cell (10^9^/L)	10.61	9.83	14.33	/	12.76	/
Blood neutrophil (10^9^/L)	8.89	8.90	11.92	/	10.81	/
Blood hemoglobin (g/L)	109	112	92	/	88	/
Blood platelet count (10^9^/L)	274	276	114	/	332	/
C-reactive protein (mg/L)	> 90	244	22.2	/	66.9	/
Procalcitonin (ng/mL)	0.39	0.33	0.36	/	0.11	/
Serum aspartate aminotransferase (U/L)	15	12	19	11	15	24.5
Serum alanine aminotransferase (U/L)	11	10	4	10	21	27.5
Serum albumin (g/L)	/	32.3	35.2	35.4	34.7	29.2
Total bilirubin (µmol/L)	/	5.8	5.1	6.5	6.5	5.0
N-terminal pro-B-type natriuretic peptide (pg/mL)	327	431	431	/	/	/
High-sensitivity Troponin T (ng/L)	18.1	23.3	19.4	/	32.9	/
**Data of Cr and CysC**						
Serum Cr (mg/dL)	6.14	5.86	7.54	4.33	5.26	0.65
Serum CysC (mg/L)	/	1.73	1.88	2.16	/	1.63
Serum Cr/CysC (L/dL)	/	3.39	4.01	2.00	/	0.40
Urine output (mL/d)	0	0	0	0	1510	1150
Urinary Cr (mg/dL)	/	/	/	/	/	81.8
Urinary CysC (mg/L)	/	/	/	/	/	0.35
Ascites Cr (mg/dL)	/	/	/	10.19	11.80	0.60
Ascites CysC (mg/L)	/	/	/	/	1.8	2.15

Cr: creatinine; CysC: Cystatin C.

Subsequent to treatment, the patient’s vital signs stabilized, and abdominal discomfort subsided. However, anuria persisted, and ascites continued to be drained daily. Further examinations, which included ultrasound imaging of the liver, gallbladder, pancreas, spleen, and portal vein, aimed to eliminate common ascites causes, such as portal hypertension and constrictive pericarditis. Biochemical assessment of the ascites disclosed an ascites creatinine level of 11.8 mg/dL and an ascites creatinine-to-blood creatinine ratio exceeding 2 (Table 1), raising suspicion of urinary ascites. A Foley catheter was inserted, followed by the instillation of a methylene blue solution into the bladder; this revealed a positive methylene blue test by contrasting the ascites’ color (Fig. 1.D).

Post-Foley catheter insertion, the patient’s urine output reverted to normal levels (1510 mL/19 h), abdominal distension diminished, and ascites substantially declined. The dialysis catheter was subsequently removed. After three days, serum creatinine decreased to 0.65 mg/dL, and serum CysC measured 1.63 mg/L. The serum creatinine/CysC ratio was 0.4 L/dL, suggesting reduced muscle mass as lean tissue index of 11.5 kg/m^2^ in the body composition at admission. Concurrently, urine CysC was detected at 0.35 mg/L, urine creatinine at 81.8 mg/dL, ascites creatinine at 0.60 mg/dL, and ascites CysC at 2.15 mg/L. The ascites creatinine/blood creatinine ratio normalized. Cystoscopy revealed inflamed and congested bladder mucosa. Contrast-enhanced fluid containing a radio-opaque agent was infused into the bladder via the catheter, and cystography examination disclosed no leakage (Fig. 1.E). Upon returning to the ward, methylene blue was reintroduced into the bladder, resulting in a negative methylene blue test by contrasting the ascites’ color, signifying the healing of the bladder wall defect. The Foley catheter was removed 25 days later, and the patient experienced no recurrence of abdominal pain. Renal function remained stable during follow-up assessments.

## Discussion and conclusions

Urinary ascites can be caused by traumatic or spontaneous bladder rupture. The diagnosis of the latter may be delayed due to its insidious onset [1]. Reactive injury of the bladder caused by alcoholism, thinning of the bladder wall due to urinary obstruction, and other similar factors commonly lead to spontaneous bladder rupture [5]. Risk factors that increase the likelihood of it include a history of pelvic malignancy radiotherapy [6], having undergone previous pelvic surgery [7], blunt pelvic trauma, alcohol intoxication [8], continuous bladder irrigation, delivery [9], drug abuse [10], and bladder diverticulum [11], among others [12]. The clinical manifestations of this condition involve acute abdominal pain, ascites, anuria or oliguria, and AKI [4]. As urine seeps through the abdominal cavity, its high creatinine content is processed through peritoneal dialysis and causes a marked rise in the patient’s blood creatinine levels, although their renal functions are not substantially impaired. Thus, urinary ascites is a type of pseudo-AKI.

Peritonitis symptoms may occur concurrently with urinary ascites, which can result in misdiagnosis as infection-related AKI or hepatorenal syndrome, culminating in delayed or even missed diagnosis. In Mokoena’s review of 44 cases [13], the average time from admission to diagnosis was 5.4 days, with a range of 2 h to 36 days. Over the past two decades, occurrences of this condition with delayed diagnoses have been frequently reported (Table 2). The reasons for delayed diagnosis in this case include (1) early suspicion of contrast-induced nephropathy and infection-related AKI caused by prior contrast agent administration and peritonitis in the presence of pre-existing chronic kidney disease, and (2) the exclusion of creatinine from routine ascites biochemical tests.

**Table 2:** The diagnosis and treatment of adult urinary ascites in recent 20 years

Year	Arthor	Age	Gender	Risk factors	Diagnosis	Delayed diagnosis (d)	Treatment
2009	Hassan [14]	39	Female	History of cesarean section	Cystography, ACr/SCr	4	IC
2011	Al-Mandeel [7]	19	Female	History of left ovariectomy	Laparotomy	NA	SR
2011	Dahiya [15]	28	Female	History of left ovariectomy	Cystography, ACr/SCr	8	IC
2011	Ko [9]	37	Female	History of salpingectomy	NA	NA	IC
2011	Aber [16]	47	Female	Paralysis with aphasia	Laparotomy	NA	SR
2012	Ridinger [17]	41	Female	Drink heavily	ACr/SCr	12	IC
2013	Arunkumar [18]	36	Female	History of cholecystectomy	ACr/SCr	NA	IC
2014	Jairam [19]	38	Male	Drink heavily	Cystography, ACr/SCr	NA	IC
2015	Matsumura [12]	63	Male	History of transurethral cystectomy	ACr/SCr	NA	IC
2017	Dawkins [20]	50	Female	History of hysterectomy	Cystoscopy	4	SR
2019	Ilktac [21]	48	Female	History of rectotomy	ACr/SCr	NA	IC, SR
2018	Wang Liang [22]	57	Male	Obstruction of lower ureter	ACr/SCr	NA	Left nephrostomy
2023	This study	85	Female	Abdominal radiotherapy	Methylene blue test, ACr/SCr	23	IC

Note: Searched in Pubmed database, with urinary ascites/urinary ascites as title/keyword to screen medical records from 2002 to 2022, excluding patients with trauma history. ACr/SCr: ascites creatinine /serum creatinine; CR: creatinine; IC: indwelling catheter; NA: not available; SR: surgical repair

Currently, there are no non-invasive or simple detection methods for diagnosing urinary ascites. In the past, ascites creatinine levels in relation to blood creatinine levels have been used as a diagnostic measure, with urinary ascites being diagnosed with an ascites creatinine/blood creatinine ratio > 2 [7, 12, 14, 17–19, 21]. However, this indicator entails obtaining and testing ascitic fluid samples. Retrograde cystography can diagnose bladder rupture and assess whether bladder repair or catheterization is effective, but it cannot provide rapid bedside diagnosis. Additionally, although bladder ultrasound corroborates the diagnosis of urinary ascites through urine flow at the site of bladder rupture, its sensitivity remains unclear [23]. In this particular case, methylene blue testing was used to diagnose the condition, which is slightly simpler than retrograde cystography. To prevent misdiagnosis of pseudo-AKI caused by urinary ascites, we recommend that ascitic fluid creatinine screening be included in the routine tests of patients with AKI and ascites.

We posit that an increase in serum creatinine while serum CysC remains stable (or a considerable increase in the ratio of serum creatinine to CysC) may indicate false AKI caused by urinary ascites and aid in early clinical diagnoses. Serum CysC is a cysteine protease inhibitor with a stable production rate in most nucleated cells. With a low molecular weight (13.3 kDa), it is mainly reabsorbed in the proximal tubules after free filtering in the renal glomerulus, leading to low concentration in urine [24, 25] (the patient in this case had a urine CysC concentration of 0.3 mg/L, < 0.28 mg/L in normal people [25]). In patients with urinary ascites, even if urine CysC enters the abdominal cavity, it has minimal effect on the concentration of CysC in the abdominal fluid and blood. Conversely, urinary creatinine concentrations are tens of times higher than blood creatinine (the patient’s urine creatinine concentration was 81.8 mg/dL) and can significantly raise serum creatinine levels by peritoneal dialysis when it enters the abdominal cavity. As a result, in patients with urinary ascites, serum creatinine may increase while CysC remains stable, or the serum creatinine/CysC ratio may exceed typical levels. Once a urinary catheter is inserted in the patient, the blood creatinine levels return to baseline, and the serum creatinine/CysC ratio also goes back to the normal range (0.4 L/dL). Our prior cross-sectional analysis reported that the 99th percentile upper limit of serum creatinine/CysC in patients with chronic kidney disease was 1.54 L/dL [26], and other studies indicated that serum creatinine/CysC is less than 2 L/dL except some extreme values [27–29]. In this case, the serum Cr/CysC ratio exceeded 2 L/dL thrice, suggesting that urinary ascites and false AKI could be possibilities. Additionally, we thought serum Cr/CysC may recognize other types of pseudo-AKI [30, 31]. But further research would be necessary to confirm it.

The treatment options for spontaneous bladder rupture comprise catheterization or surgical repair. For small bladder ruptures, catheterization would suffice without the need for further intervention, and the bladder eventually repairs itself through omentum wrapping or self-contraction. For larger ruptures (diameter exceeding 7 mm according to literature), surgical repair may be advisable. Previous reports indicate that catheterization is a satisfactory management option for most cases [9, 12, 14, 15, 17–19]. However, there is a reported exception where catheterization failed to heal the condition, and surgery was necessary [21]. Nevertheless, surgical repair has a good record of efficacy [7, 16, 20].

In summary, this case highlights the delayed diagnosis of urinary ascites with pseudo-AKI in a female patient with a history of pelvic radiotherapy and urinary retention. It underscores the importance of routinely screening ascites creatinine in patients with unexplained ascites and creatinine-based AKI. Furthermore, an increase in serum creatinine without a corresponding increase in serum CysC (or an increased ratio of blood creatinine to blood CysC) may indicate pseudo-AKI due to urinary ascites. Further investigations are warranted to evaluate the diagnostic utility of serum creatinine/CysC ratio for urinary ascites and pseudo-AKI.

## Data Availability

Not applicable as no data was used in this article.

## List of abbreviations

Cr: Creatinine
CysC: Cystina C
AKI: acute kidney injury
